# A development study for liquid- and vapor-fed anode zero-gap bioelectrolysis cells

**DOI:** 10.1016/j.isci.2025.112959

**Published:** 2025-06-19

**Authors:** Nils Rohbohm, Largus T. Angenent

**Affiliations:** 1 Environmental Biotechnology Group, Department of Geosciences, University of Tübingen, Schnarrenbergstraße 94-96, 72076 Tübingen, Germany; 2 Cluster of Excellence – Controlling Microbes to Fight Infections, University of Tübingen, Auf der Morgenstelle 28, 72076 Tübingen, Germany; 3 AG Angenent, Max Planck Institute for Biology Tübingen, Max-Planck-Ring 5, 72076 Tübingen, Germany; 4 Department of Biological and Chemical Engineering, Aarhus University, Gustav Wieds Vej 10D, 8000 Aarhus C, Denmark; 5 The Novo Nordisk Foundation CO_2_ Research Center (CORC), Aarhus University, Gustav Wieds Vej 10C, 8000 Aarhus C, Denmark

**Keywords:** Electrochemistry, Bioengineering, Biotechnology

## Abstract

Improving microbial electrosynthesis could be one solution for transitioning toward sustainable chemical production, offering a pathway to convert CO_2_ into valuable commodities from renewable energy sources. Therefore, we further developed liquid- and vapor-fed anode zero-gap bioelectrochemical cells for electromethanogenesis, utilizing a membrane electrode assembly to enhance mass and ohmic transport. Focusing on CH_4_ and H_2_ production, we tested two ion-exchange membranes with the liquid-fed anode system and selected the best-performing ion-exchange membrane for the vapor-fed anode system. The liquid-fed anode system did not show considerable differences in volumetric CH_4_ production rates compared to vapor-fed anode systems. However, the latter demonstrated advantages in reducing electrocatalyst degradation and maintaining stable cell voltages, resulting in the highest reported maximum CH_4_ production efficiency of 48.7 L kWh^−1^, thus far. The research underscores the need for further optimization to address performance losses and suggests potential for industrial applications of microbial electrosynthesis, highlighting the importance of catalyst protection.

## Highlights

• Liquid- and vapor-fed anodes for zero-gap bioelectrochemical cells were compared
• A vapor-fed anode zero-gap cell achieved a CH_4_ production efficiency of 48.7 L kWh^−1^
• Vapor-fed anode operation maintained lower cell voltages and ohmic resistances
• Adding a PTFE membrane offered modest improvements

## Graphical abstract

**Figure:**
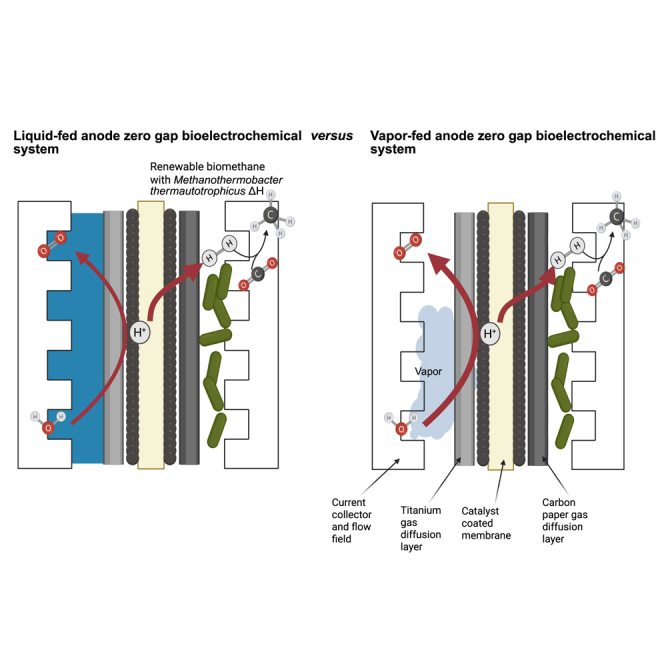

## Introduction

Converting electrical energy into chemical energy carriers, such as methane gas (CH_4_), through the synergy of electrochemistry and biology^1^ holds the potential to mitigate major greenhouse gas emissions while facilitating the storage of renewable energy. The amalgamation of these steps occurs within bioelectrochemical cells, defining the field as microbial electrochemistry.^2^ The specific term for the CO_2_ upgrading at the biocathode to CH_4_ is electromethanogenesis. Furthermore, integration could streamline the process economically by eliminating the need for abiotic electrolysis as a separate unit operation. Integrated bioelectrochemical cells have promising advantages compared to other abiotic electrosynthesis systems, such as CO_2_ electrolyzers, which rely on metal catalysts. These benefits are especially important given the challenges abiotic systems face with electrocatalyst selectivity, stability, material availability, and cost.^3^

Biomethanation through gas fermentation achieves much higher production rates than microbial electromethanogenesis.^4^ In fact, the development of electromethanogenesis is currently constrained by low current densities, which remain the primary bottleneck. Several studies have aimed to enhance these current densities by modifying the cathode, optimizing reactor design, or promoting cathodic biofilm formation.^5^^,^^6^^,^^7^ Although improvements in methane production rates have been reported, current densities remain insufficient for commercial viability (Table S1). Notably, energy efficiency has reached up to 43%,^8^ whereas the highest reported CH_4_ production efficiency (Table S1, 42.9 L kWh^−1^) was achieved using a bubble column reactor with a 3D-printed NiMo-coated cathode.^9^

Although alternative reactor designs have achieved high current densities, these advancements often come at the cost of considerably increased cell voltages (Table S1).^10^^,^^11^ Regarding current density improvement with low cell voltage, the zero-gap design is one promising direction. Geppert and colleagues achieved a breakthrough by implementing the electrochemical design of a redox-flow battery, achieving a current density of 3.5 mA cm^−2^ for one day with approximately 30% energy efficiency in CH_4_ production.^12^ Deutzmann et al. achieved 6.3 mA cm^−2^ at 3 V using a flat-plate reactor with a high-surface-area cathode (Table S1).^13^ In comparison, Rad et al. reported a breakthrough current density of 30 mA cm^−2^ at 2.1 to 2.3 V using a hybrid bioelectrochemical system, incorporating a hydrophobic porous transport layer to isolate the cathodic catalyst from microbial contact.^8^ Scaling this zero-gap architecture by stacking individual cells could enable industrial applications, following strategies used in electrolyzers and fuel cells.

For an efficient electrochemical cell, it is imperative to protect the electrocatalysts from contaminants. While abiotic liquid-fed anode electrolyzers necessitate ultra-pure water not to degrade the electrocatalyst, vapor-fed anode electrolyzers only need humid air to resolve a possible source of contamination.^14^ This simplifies the overall setup and allows the application of electrolyzers in areas where clean water is not readily available. Using contaminated water or seawater that exhibits performance issues with liquid-fed anode electrolyzers would also be possible.^15^ A bioelectrochemical system with a vapor-fed anode was developed to maintain CH_4_ production for several weeks at 1.7 mA cm^−2^.^16^ The relatively low current density was achieved at a high cell voltage (2.8 V), and further improvements are warranted before commercial viability.

Here, we built on this zero-gap and vapor-fed anode design to assess their suitability for electromethanogenesis. We performed an engineering development study to focus on how membrane selection and anode configuration influence catalyst degradation. We also focused on the operating trade-offs between liquid- and vapor-fed systems. First, we evaluated two ion-exchange membranes in a liquid-fed anode zero-gap cell, followed by testing a vapor-fed configuration using the optimal membrane. We then evaluated the performance of both systems. Finally, we investigated the impact of adding a PTFE membrane to the vapor-fed cell to determine its effectiveness in shielding the cathode catalyst from fermentation broth exposure.

## Results

### Htec-PFSA membrane underperformed compared to the Nafion 117 membrane in liquid-fed cells

One of our aims was to increase the efficiency of bioelectrochemical systems for electromethanogenesis, with a particular focus on optimizing the performance of liquid-fed anode zero-gap cells. We evaluated the effectiveness of two distinct catalyst-coated ion-exchange membranes—Htec-PFSA (perfluoro sulfonic acid) and Nafion 117—within the affordable, commercially available cell that we utilized (see STAR Methods for details of the catalyst-coated membranes [CCMs]), aiming to ascertain which membrane facilitates electromethanogenesis best. The goal was to identify the best-performing membrane, considering the differences in catalyst type and concentration (see STAR Methods, bioelectrochemical Setup), for subsequent use in the vapor-fed anode zero-gap cell.

The experimental procedure involved operating one bioelectrochemical cell as a liquid-fed anode, zero-gap, incorporating one of the two selected membranes. The bioelectrochemical cell was operated in a fed-batch mode, receiving a continuous supply of CO_2_ as the only carbon substrate for electromethanogenesis. By employing chronopotentiometric control, we ensured a consistent electric current of 120 mA across the cell, achieving a geometric current density of 7.5 mA cm^−2^. Preliminary findings (not included in this study) had already indicated that such a current density initially produced a cell voltage below 2 V, aligning with one criterion for efficiency. The reactor started with an optical density (OD_600_) of 0.04 after inoculation with *Methanothermobacter thermautotrophicus* ΔH. The inoculation process was performed once to reduce the experimental error by using the same bioelectrochemical cell with the same active pure culture. First, the reactor was inoculated with the Htec-PFSA membrane already been installed. Next, the same pure culture was utilized for the Nafion 117 membrane by replacing the catalyst-coated membrane (CCM).

**Figure 1:**
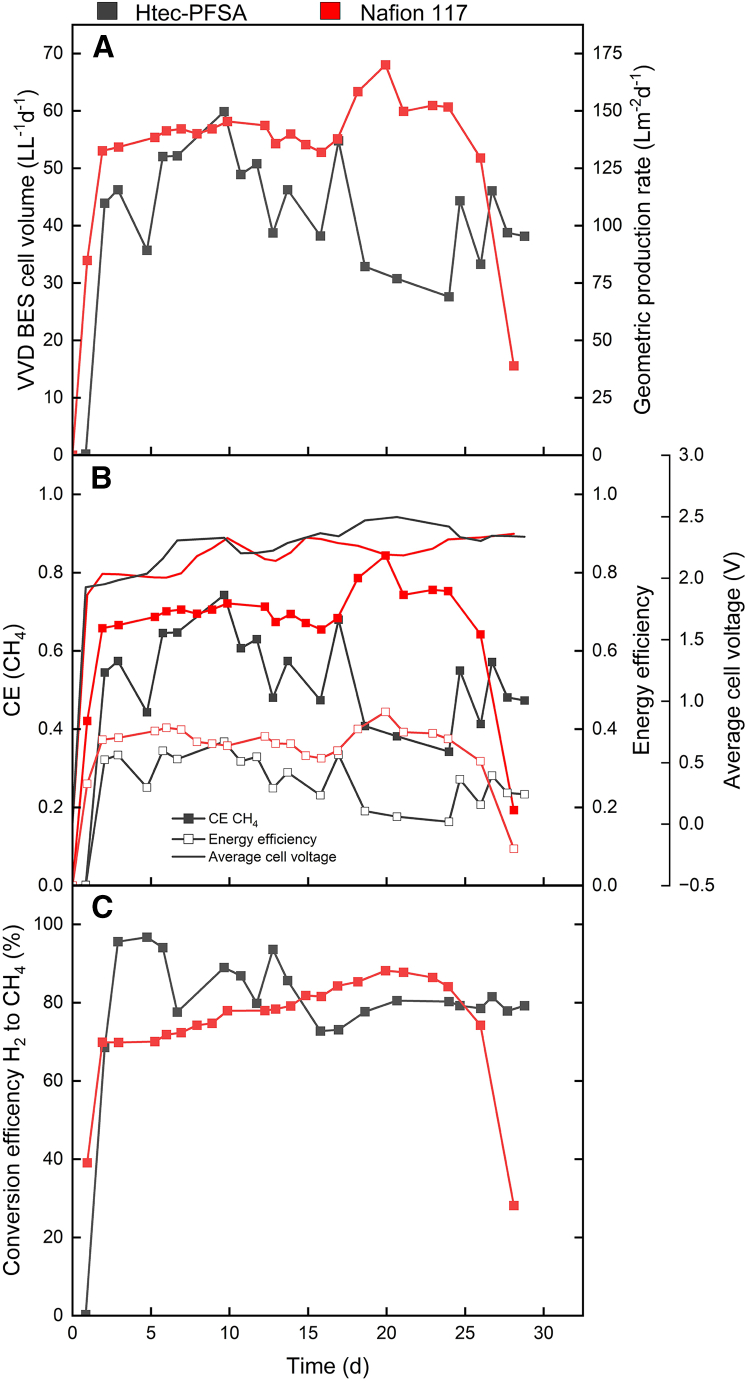
Results from an experiment with a liquid-fed anode zero-gap bioelectrochemical cell using two ion-exchange membranes, Htec-PFSA and Nafion 117, which are coded in dark gray and red, respectively The different graphs show (A) the volumetric (Y1-axis) and geometric (Y2-axis) CH_4_ production rate; (B) the CEs (Y1-axis), energy efficiencies (Y2-axis), and average cell voltage (Y3-axis); and (C) the conversion efficiency of H_2_ to CH_4_. The sudden performance drop for the bioelectrochemical cell using Nafion 117 corresponded to a leakage after which we ended the experiment.

After inoculating the zero-gap bioelectrochemical cell, a brief one-day lag phase preceded the initiation of electromethanogenesis (Figure 1). Notably, the cell exhibited efficient H_2_ production, achieving an H_2_ Coulombic efficiency (H_2_-CE) of 94.3% (Figure S1). Commencing the post-lag phase, the volumetric CH_4_ production rate (i.e., volume of CH_4_ per bioelectrochemical cell catholyte volume per day) peaked at 59.9 L L^−1^ d^−1^ by day 10 (Table S2), which was accompanied by a 74.3% CH_4_ Coulombic efficiency (CH_4_-CE) (Figures 1A and 1B). Overall, the reactor had a stable phase, displaying minor fluctuations until day 17, maintaining an average volumetric CH_4_ production rate of 47.3 L L^−1^ d^−1^ at an average CH_4_-CE of 58.7%. The overall average volumetric CH_4_ production rate throughout the 28-day experimental period, excluding the lag phase, was 43.0 L L^−1^ d^−1^ with an average CH_4_-CE of 50.8%. The maximum energy efficiency (EE) obtained was 36.8% (Figure 1B).

After changing the CCM to Nafion 117, we did not observe a noticeable lag phase in methane production (Figure 1A). The system demonstrated efficient volumetric CH_4_ production, attaining a peaked volumetric CH_4_ production rate at 68.0 L L^−1^ d^−1^ on day 20 (Table S2), with a CH_4_-CE of 84.4% and a 44.4% EE (Figures 1A and 1B). The system had a stable production phase with minor fluctuations, maintaining an average volumetric CH_4_ production rate of 56.0 L L^−1^ d^−1^ at an average CH_4_-CE of 69.4%, excluding day 28 when the cell leaked (drop of volumetric CH_4_ production rate, Figure 1A), which also finalized the experiment. Comparing the performance of Htec-PFSA and Nafion 117 ion-exchange membranes for volumetric CH_4_ production revealed an advantage for Nafion 117. The performance of Nafion 117 surpassed the one of Htec-PFSA membrane, showing an 11.9% higher peak volumetric CH_4_ production rate and a 23.1% increase in the average volumetric CH_4_ production rate. Furthermore, the H_2_-CE fluctuated more during the Htec experiment than during the Nafion 117 experiment, revealing differences between the membranes. This outcome suggests that Nafion 117 has an advantageous application compared to the Htec-PFSA membrane in this study. We hypothesized that the cell exhibited greater stability with the Nafion 117 system, which was further explored in the subsequent section.

### Nafion 117 membrane exhibited better stability and efficiency compared to the Htec-PFSA membrane, evidenced by lower increases in cell voltage and better H_2_ production efficiency

One of the flaws in bioelectrochemistry is the lack of protection of the electrocatalysts, which are susceptible to degradation throughout time due to harsh environmental conditions. These conditions often involve exposure to a medium that is enriched with metals, which are essential for microbial growth, adversely affecting the integrity of the electrocatalysts. While anode degradation can be somewhat mitigated through the utilization of pure water, the inevitable wear and the potential crossover of ions from the cathode to the anode chamber can compromise the performance of anodic electrocatalysts. Still, a predominant source of catalyst degradation is attributed to the catholyte, which contains the growth medium for the microbes. Here, the electrocatalyst was Pt/C, which is particularly vulnerable to poisoning by sulfides present in the medium. A key indicator of this degradation is the increase in the cell voltage needed to sustain a constant applied current, where an increase in cell voltage is inversely related to the activity of the catalyst. During the liquid-fed anode experimental periods, both the Htec-PFSA and Nafion 117 membranes exhibited a decline in catalytic activity, shown by an increase in cell voltages. Specifically, the Htec-PFSA membrane experiment recorded a cell voltage rise from 1.92 to 2.49 V (Figure 1B). Conversely, the setup with the Nafion 117 membrane demonstrated a more modest increase, from 1.86 to 2.36 V. This smaller increase in cell voltage, which we observed with the Nafion 117 membrane, is an advantage for the H_2_ production efficiency. However, given the lack of replicates, this difference is not statistically significant and not substantial enough to establish a definitive advantage of one membrane compared to the other in terms of cell voltage. It is an indication, though, that further work can commence with Nafion 117.

The ideal scenario involves producing H_2_ without byproducts or losses. However, as cell voltages increase, there is a discernible decrease in catalytic activity through catalyst degradation, increased overpotentials, possible side reactions, and loss of selectivity, impacting H_2_ production. The Htec membrane exhibited a higher cell voltage than Nafion 117, leading to a steady decline in H_2_-CE and limiting CH_4_ production. Conversely, Nafion 117 demonstrated a more stable H_2_-CE at 91.2% versus 66.5% for the Htec membrane (Figure S1). While both membranes experienced periods of elevated cell voltages, the bioelectrochemical cell with Nafion 117 had an overall lower cell voltage throughout the experiment compared to the cell using the Htec-PFSA membrane, resulting in less disruption to H_2_ production. Notably, at an average cell voltage of 2.47 V, the H_2_-CE of the Htec membrane crashed (Figure S1). While the Htec membrane demonstrated a higher conversion efficiency of available H_2_ to CH_4_ at 82.4% compared to 76.6% of Nafion 117 (Figure 1C), the Nafion 117 setup outperformed overall due to its higher volumetric CH_4_ production rate (Table S2) and H_2_-CE. As a consequence, the vapor-fed anode experiment was conducted with the membrane electrode assembly using the Nafion 117 ion-exchange membrane.

### Electromethanogenesis in a vapor-fed anode zero-gap bioelectrochemical cell was not influenced by pressure adjustments in the anodic chamber, but H_2_ production was

**Figure 2:**
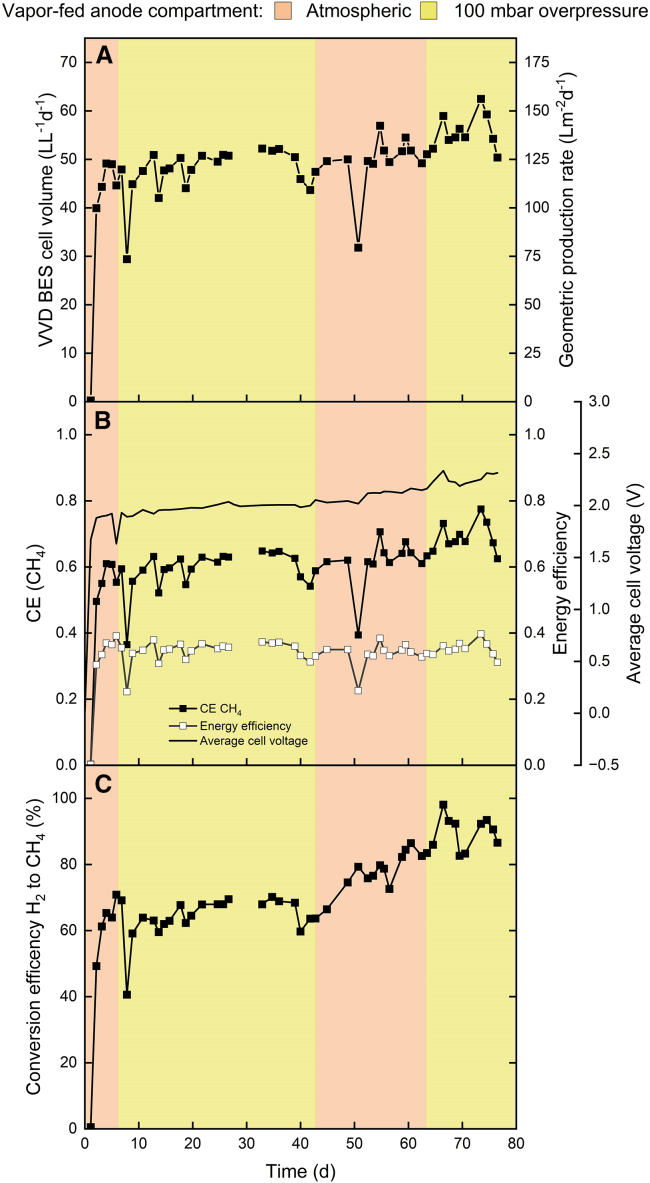
Results from an experiment with a vapor-fed anode zero-gap bioelectrochemical cell using Nafion 117 The anode compartment of the bioelectrochemical cell was kept at atmospheric pressure (orange) or 100 mbar overpressure (yellow). The different graphs show (A) the BES cell volumetric (Y1-axis) and geometric (Y2-axis) CH_4_ production rate; (B) the CEs (Y1-axis), energy efficiencies (Y2-axis), and average cell voltage (Y3-axis); and (C) the conversion efficiency of H_2_ to CH_4_. The decrease in H_2_ production (Figure S2) decreased the excess H_2_ in the system, which resulted in the steady increase in the conversion efficiency of H_2_ to CH_4_.

Here, a vapor-fed anode zero-gap bioelectrochemical cell with Nafion 117 was operated in the same manner as for the liquid-fed anode experiments. The introduction of vapor into the anode chamber was controlled at 3 mL min^−1^, following a 10-min equilibration period, to ensure system stability before commencing measurements. The experiment started with an OD_600_ of 0.04 after inoculation with *M. thermautotrophicus* ΔH. Similar to the liquid-fed anode experiment utilizing the Htec-PFSA membrane, a one-day lag phase was observed before the onset of electromethanogenesis. During the initial 5 days, without pressurization at the anode, the cell averaged a volumetric CH_4_ production rate of 45.6 L L^−1^ d^−1^, peaking at 49.1 L L^−1^ d^−1^ (Figure 2A). The CH_4_-CE averaged 56.6%, reaching a maximum of 60.9% (Figure 2B). This performance was comparable to that observed with the Htec membrane experiment for the liquid-fed anode.

To address the issue of back pressure from the cathodic chamber and to reduce H_2_ crossover, the anode chamber was subsequently pressurized to 100 mbar on day 6 of the experimental period. This adjustment maintained an average volumetric CH_4_ production rate of 47.4 L L^−1^ d^−1^ with an average CH_4_-CE of 58.8%. The peak volumetric CH_4_ production rate and CH_4_-CE were 52.2 L L^−1^ d^−1^ and 64.8%, respectively. The highest EE was 39.1% on day 7. On day 43, the pressure from the anodic compartment was released again to atmospheric pressures to evaluate the impact on the performance. Following the release of pressure, the average H_2_-CE was 91.4%, and the H_2_-to-CH_4_ conversion efficiency was 65.5% (Figures 2C and S2), suggesting that H_2_ availability was not a limiting factor, with primary losses attributed to outgassing. Despite the initial high H_2_-CE of 92.7%, it declined to 75.9% by the end of the observation period, averaging 78.7% (Figure S2). Meanwhile, the volumetric CH_4_ production remained steady at 49.7 L L^−1^ d^−1^. Thus, adding pressure to the anode during the fed-batch mode did not substantially change the performance.

On day 51, a sharp drop in CH_4_ production occurred due to an unknown reason. By chance, it coincided with the high observed optical density (O.D.) of 0.85 (Figure S3). Still, we were worried that the drop was due to nutrient limitations, and we switched to continuous operation for the catholyte at a hydraulic retention time of 7 days on day 52 (until day 80). The continuous mode decreased and then stabilized the O.D. and improved performance. During continuous operation, increasing the pressure to 100 mbar on day 62 slightly increased the average volumetric CH_4_ production rate, averaging 55.7 L L^−1^ d^−1^ with a peak of 62.5 L L^−1^ d^−1^ and EE of 39.7% (Table S2). We observed the most considerable change in performance for the H_2_-to-CH_4_ conversion efficiency, which rose from 80.3% pre-pressurization to 89.8% post-pressurization (Figure 2C). However, we attribute this to a lower H_2_ production rate and a reduced excess of H_2_ in the headspace. Therefore, this latest pressurization at continuous operation did not really improve electromethanogenesis, which can be best seen from the flat energy efficiency curve during all pressurization and depressurization events (Figure 2B).

### Vapor-fed anode zero-gap bioelectrochemical cell maintained a lower cell voltage and reduced electrocatalyst degradation

One of the reasons that a bioelectrochemical system with a vapor-fed anode was developed was to circumvent a pH gradient,^16^ which would otherwise contribute to an elevated thermodynamic cell voltage. Additionally, this approach considerably reduces the degradation of the electrocatalyst, which is a notable advantage compared to the liquid-fed anode system, where electrocatalyst degradation is more pronounced. In comparison, the liquid-fed anode bioelectrochemical cell demonstrated a marked increase in cell voltage, from 1.86 to 2.36 V throughout 28 days (Figure 1B). In contrast, the vapor-fed anode bioelectrochemical cell exhibited voltage stability, maintaining a more stable cell voltage of 1.96 V for 42 days (Figure 2B). This stability persisted until the pressure was released from the anode compartment. Subsequent to the pressure release, the cell had a decline in H_2_ production efficiency accompanied by an increase in cell voltage, which peaked at an average maximum of 2.33 V (Figure 2B). The stability of the catalyst, which is a factor in the long-term performance of bioelectrochemical systems, was assessed by monitoring the change in cell voltage throughout the operating period and was 172 μV h^−1^.

**Figure 3:**
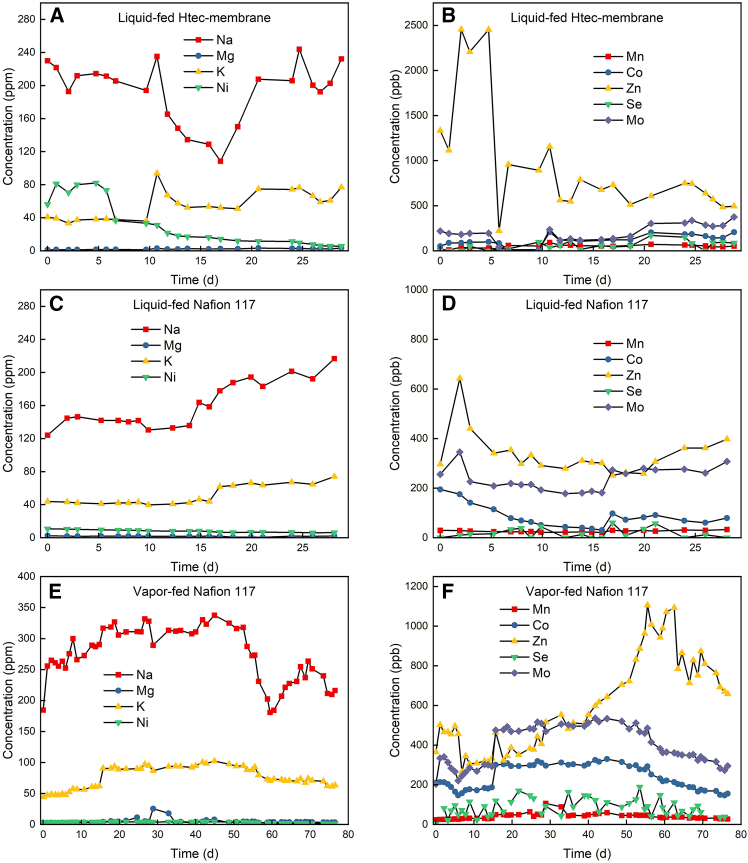
Element concentrations Na, Mg, K, Ni, Mn, Co, Zn, Se, and Mo in the catholyte measured with ICP-MS during the liquid- and vapor-fed anode bioelectrochemical experiments (A and B) show the liquid-fed experiment using the Htec-PFSA membrane, (C and D) demonstrate the liquid-fed anode experiment using the Nafion 117 membrane, and (E and F) exhibit the vapor-fed anode experiment using the Nafion 117 membrane.

The degradation of catalysts can change the resistance of the electrolyzer. We evaluated the ohmic resistance involving various contributors such as contact resistance, catalyst resistance, and solution resistance. We employed the current interrupt method to measure the ohmic resistance of the bioelectrochemical cell because inserting a reference electrode for impedance measurements in the bioelectrochemical cell was not feasible. An increase in the bioelectrochemical cell voltage of 500 mV theoretically indicates a rise in ohmic resistance. Our findings corroborate this theory: the initial area-specific ohmic resistance was measured at 18 ± 0.46 Ω cm^2^ at the onset of the experiment and increased to 28 ± 0.10 Ω cm^2^ by its conclusion, which is a 1.5-fold increase. This rise in area-specific ohmic resistance suggests that the membrane electrode assembly had become less efficient. Given that the electrolyte composition remained unchanged (Figure 3), suggesting minimal impact from solution resistance, we hypothesize that the catalyst and contact resistance are the primary contributors. Furthermore, prolonged exposure to high cell voltages may have also contributed to membrane deterioration and higher resistance. The liquid-fed anode zero-gap bioelectrochemical cell exhibited an area-specific ohmic resistance of 34 ± 0.33 Ω cm^2^, which was 1.2 times higher at an operation of 28 days versus 76 days for the vapor-fed anode system. In conclusion, vapor-fed anode bioelectrochemical cells offer advantages in terms of voltage stability and reduced electrocatalyst degradation compared to liquid-fed anode bioelectrochemical systems.

### Metal compound dynamics had no substantial impact on liquid- and vapor-fed anode bioelectrochemical experiments

The zero-gap bioelectrochemical cell experiments observed a consistent decline in H_2_ production, concurrently impeding CH_4_ conversion. This reduction in H_2_ production indicates reduced electrocatalytic activity at the cathode. Alongside, increasing cell voltage across all experiments further suggests catalysts deactivation. The possibility of cathode poisoning via electroplating of metal compounds is considered, which was shown by another study that electroplated metal compounds onto cathodes using concentrated trace element solutions.^5^ Consequently, in our study, analytical measurements of various elements, namely ^23^Na, ^24^Mg, ^39^K, ^55^Mn, ^59^Co, ^60^Ni, and ^95^Mo, were conducted (Figure 3). No deficiencies in the medium were noted, and element concentrations remained stable throughout the experiments. Notably, despite no initial deficiency, a decline in ^60^Ni concentration was observed, suggesting an increased uptake by *M. thermautotrophicus* ΔH compared to other elements. Nickel is used in the Ni-enzymes of *M. thermautotrophicus* ΔH to enhance growth.^17^^,^^18^ Other possible causes of nickel decline include precipitation or electroplating. In all three cases, we lack substantial evidence to determine which has the greatest impact. Overall, the vapor-fed anode bioelectrochemical system is the optimal setup for this study. Still, measures should be taken to prevent element deficiencies during the operation, especially from nickel and magnesium.

### A PTFE membrane, which was placed before the cathode, only gave a small advantage in protecting the catalysts layer

The vapor-fed anode zero-gap configuration improved stability and longevity by minimizing anode deterioration, leading to a more stable cell voltage and reduced resistance fluctuations compared to the liquid-fed setup. In this subsequent experiment, the vapor-fed anode zero-gap bioelectrochemical cell had an additional PTFE membrane in front of the cathode catalyst. One of the key challenges in bioelectrochemical systems is the degradation of the cathode catalyst due to exposure to the fermentation broth, which can lead to reduced system performance, higher energy consumption, and faster catalyst degradation. By placing a PTFE membrane in front of the cathode, the aim was to create a physical separation between the cathode and the fermentation broth, reducing direct contact and thus protecting the catalyst, similar to Rad et al.^8^ However, the results demonstrated that the PTFE membrane did not offer a considerable protective advantage, although it did impact other operating parameters.

**Figure 4:**
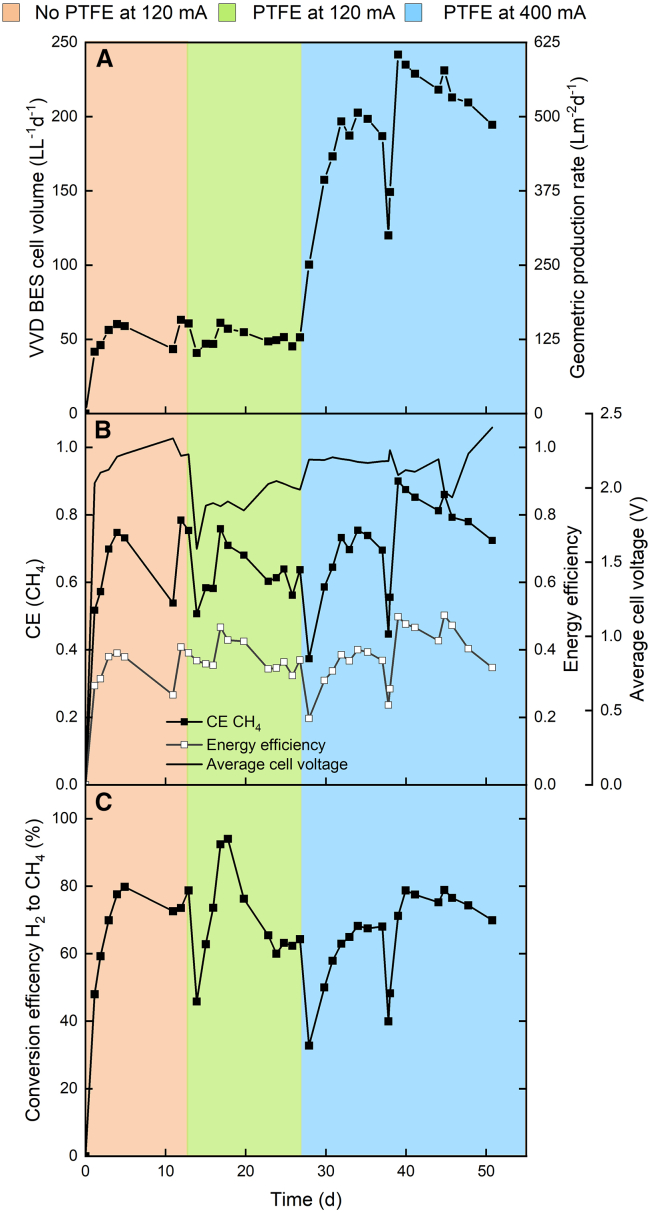
Zero-gap bioelectrochemical vapor-fed anode experiment using Nafion 117 The orange phase represents the initial operation of the bioelectrochemical cell without a PTFE membrane, whereas the green phase signifies the start of operation with the PTFE membrane added. In the blue phase, the current was increased from 120 to 400 mA with the PTFE membrane in place. The different graphs show (A) the BES cell volumetric (Y1-axis) and geometric (Y2-axis) CH_4_ production rate; (B) the CEs (Y1-axis), energy efficiencies (Y2-axis), and average cell voltage (Y3-axis); and (C) the conversion efficiency of H_2_ to CH_4_.

Initially, the bioelectrochemical cell was operated for 13 days without a PTFE membrane (Figure 4), under conditions identical to the previous experiment (Figure 2). The vapor feeding was increased to ensure that enough moisture was created and to avoid pressurizing the system. During the first 13 days, the system stabilized with a CH_4_ production rate averaging 53.8 L L^−1^ d^−1^, peaking at 63.2 L L^−1^ d^−1^ (Figure 4A). The CH_4_-CE averaged 66.8%, with a peak of 78.4%, and the EE reached a maximum of 40.8% (Figure 4B). Performance was comparable to earlier experiments, showing slightly higher CH_4_ production and EE, although the cell voltage was elevated compared to the previous run. After 13 days, the bioelectrochemical cell was replaced with an identical setup containing a PTFE membrane positioned between the cathode and fermentation broth, leaving a small gap between the cathode and the PTFE membrane. With the PTFE membrane in place, the system showed a slight reduction in volumetric CH_4_ production, averaging 50.4 L L^−1^ d^−1^ and peaking at 61.1 L L^−1^ d^−1^ (Figure 4A; Table S2). The CH_4_-CE decreased to an average of 62.5%, reaching a maximum of 75.8%. However, the key observation was a reduction in the average cell voltage from 2.18 V (without the PTFE membrane) to 1.91 V (with the PTFE membrane) (Figure 4B). This voltage decrease positively influenced the EE, which peaked at 46.6% in the cell with the PTFE membrane. Compared to the experiment without the PTFE membrane, the cell voltage (average of 1.91 V) is not substantially lower and does not differ considerably. Overall, a small improvement in the cell voltage was observed but it was too small to showcase the benefit of adding a PTFE membrane.

On day 26, the current was increased from 120 to 400 mA (7.5–25 mA cm^−2^) to assess the response to higher operating stress. Along with this, the CO_2_ feed flow rate was adjusted to 0.8 mL min^−1^. As a result of the higher H_2_ availability, the volumetric CH_4_ production rate surged to 241.8 L L^−1^ d^−1^, with a CH_4_-CE of 90% (Figures 4A and 4B; Table S2). The average volumetric CH_4_ production rate and CH_4_-CE were 191.3 L L^−1^ d^−1^ and 71.2%, respectively, reflecting an improvement in CH_4_ production efficiency at the higher current. The increase in CH_4_-CE compared to the 120 mA operation highlights that raising the current can improve the efficiency in producing CH_4_. Interestingly, the fraction of H_2_ in the headspace increased at the higher current, and yet the H_2_-to-CH_4_ conversion efficiency remained similar across both current conditions (Figure 4C). This indicates that the system had more dissolved H_2_ available for CH_4_ production at the increased current, leading to improved efficiency. This increased efficiency is reflected in the higher energy efficiencies (EEs), with the maximum EE reaching 50.2% (Figure 4B; cell voltage 1.97 V, volumetric CH_4_ production rate 231.1 L L^−1^ d^−1^) on day 44. When the current was increased to 400 mA, the cell voltage initially rose from 1.98 to 2.18 V. The cell voltage remained stable until day 40, after which fluctuations occurred, with voltages exceeding 3 V being recorded (Figure S4). Such elevated cell voltages can accelerate anode catalyst degradation, making it difficult to maintain long-term voltage stability. However, when the cell voltage was stable, the EE was above 50%, suggesting that the bioelectrochemical cell with a PTFE membrane is capable of maintaining efficient performance and protecting the bioelectrochemical cell from rapid degradation, provided that the voltage is well managed.

### Abiotic tests suggest a crossover of the fermentation broth when protecting the cathode with the PTFE membrane

The goal of incorporating a PTFE membrane in the bioelectrochemical cell was to determine whether it could effectively protect the cathode from degradation while still allowing efficient electron transfer for CH_4_ production. Abiotic tests were conducted to isolate the specific impact of the PTFE membrane on the cathode, ensuring that any performance changes were not solely due to biological activity. In these tests, the bioelectrochemical cell was operated without recirculating the fermentation broth and abiotically, under applied currents of 120 and 400 mA, to evaluate how the PTFE membrane impacted the bioelectrochemical cell performance. Before starting the experiments, vapor was fed to the anode for 1 h. When a current of 120 mA was applied, the cell voltage measured 1.54 V, and at 400 mA, the cell voltage was slightly higher at 1.65 V (Figure S5). The CE-H_2_ was above 99% for all measured conditions and replicates, indicating optimal H_2_ production under these conditions.

Comparing the abiotic and biotic experiments revealed considerable differences in cell voltage. At a current of 120 mA, the voltage difference between the abiotic and biotic conditions was 0.37 V, whereas at 400 mA, the difference increased to 0.53 V. This variation is partly due to the higher thermodynamic potential difference between O_2_ and H_2_ evolution reactions, which is ideally 1.23 V. However, in this setup, the presence of a presumable pH gradient increased the thermodynamic cell voltage during the biotic experiment. Even if the PTFE membrane is hydrophobic, through convection or diffusion, H_2_O from the fermentation broth or from the ion-exchange membrane could have accumulated in the gap between the cathode and the PTFE membrane, which ideally should have contained only H_2_ gas. This accumulation could have created a subsequent pH gradient. However, there is not any substantial proof, and given the size of the gap, a collection of a sample was not possible. On day 13 of the vapor-fed anode zero-gap bioelectrochemical cell with the PTFE membrane experiment, the cell voltage was similar to the abiotic experiment at 1.52 V. On day 14, there was a sudden surge in the cell voltage to 1.88 V, marking the beginning of the supposed pH gradient. Overall, the PTFE membrane protects the cathode catalyst from the fermentation broth, but due to water convection, we assume a pH gradient forming that could be mitigated by increasing the H_2_ production or decreasing the recirculating fermentation broth pressure on the PTFE membrane.

## Discussion

The zero-gap bioelectrochemical experiments successfully ran with *M. thermautotrophicus* ΔH under two operating modes, liquid- and vapor-feeding at the anode compartment. Although both modes achieved comparable volumetric CH_4_ production rates, the vapor-fed system exhibited lower and more stable cell voltages, suggesting potential advantages for long-term operation. The liquid-fed setup reached the highest CH_4_ production rate and EE, but it also showed signs of performance degradation, indicating a trade-off between productivity and stability. Compared to a published zero-gap vapor-fed setup from Baek et al. (2022), our setup achieved higher CH_4_ production rates and operated at lower cell voltages.^16^ Similarly, our vapor-fed configuration with a PTFE membrane outperformed (in volumetric CH_4_ production rate) the system reported by Rad et al. (2023) at 30 mA cm^−2^.^8^ The maximum CH_4_ production efficiency observed in this study was 48.7 L kWh^−1^ with the vapor-fed mode, exceeding recently reported values (Tables S1 and S2). However, the volumetric CH_4_ production rate remained lower than in some previously published studies (Table S1). Therefore, it is necessary to identify the main contributors to electrocatalyst degradation and system performance in both liquid- and vapor-fed anode configurations to further optimize the zero-gap bioelectrochemical cells, providing insights for improved operation in future studies, and close the gap to gas fermentation.

When low current densities are used, H_2_ can be taken up more easily by the microbes without forming gas bubbles and outgas.^19^ Geppert and colleagues showed this observation when running a redox flow battery-type reactor, which we would classify as a zero-gap cell.^12^ They observed that the outgas concentration of H_2_ increased with higher current densities. However, H_2_ has a low solubility in water, so with higher current densities, more gas bubbles would be formed and are less likely to be taken up by the microbes. Once the diffusion layer is saturated with dissolved H_2_, H_2_ bubble formation will occur.^19^ Therefore, the absolute current that is needed to saturate the diffusion layer would be reached at low current densities. Here, bubble formation was inevitable because, assuming Fick’s law of diffusion at 60°C (with ∂ϕ = 0.75 mM and boundary thickness of 100 μm) and atmospheric pressure, the minimum current density is 1.37 mA cm^−2^ at which bubble formation would occur.^19^^,^^20^ In general, *M. thermautotrophicus* is an efficient microbe that converts H_2_ and CO_2_ at a very high conversion efficiency and yield, and Electrochaea GmbH now uses it in a commercial setting.^21^^,^^22^ Therefore, our study was not limited by the microbe. As for all gas fermentation processes, the low dissolution of H_2_ is the limiting step in efficiently converting H_2_ into any value-added chemical.^22^ Therefore, our system setup presents major flaws in efficiently retaining H_2_ to convert H_2_ and CO_2_ into CH_4_. A solution would be to pressurize the cathode to dissolve more H_2_ or to recirculate the gas phase to increase the mass transfer rate of dissolved H_2_.^10^ Increasing the surface area of the electrode would enhance the production of miscible H_2_ by providing more active sites for its generation.^7^

PFSA ion-exchange membranes are widely used in electrochemical systems. However, the use of Nafion 117 as in this study is not only one of the most studied ion-exchange membranes but also has a disadvantage. Once the ion-exchange membrane encounters stress, its performance decreases, and H_2_ crossover occurs.^23^ Here, the ion-exchange membrane can encounter swelling through exposure to higher operating temperatures, loss of cation-selectivity, mechanical stress from electrolyte recirculation, and ion precipitation on the membrane. Although the disadvantages and problems of PFSA ion-exchange membranes are known, it is unclear which disadvantages are the main reason for the performance loss of both liquid- and vapor-fed anode systems. H_2_ crossed over to the anode side (measured qualitatively) in both anodic operating modes, and we hypothesize that the swelling of the membrane is the main contributor to the H_2_ crossover. We recommend testing new ion-exchange membranes that could prevent the abovementioned disadvantages for future studies.

Furthermore, it has been shown that at higher current densities, more H_2_ crosses through the ion-exchange membrane.^24^^,^^25^^,^^26^ A contributor to H_2_ crossover is the diffusion of supersaturated H_2_. Supersaturated H_2_ is dissolved H_2_ at the boundary layer of the electrode that exceeds the average possible solubility of H_2_, which can be calculated through Henry’s law. Another potential mechanism is convection driven by pressure differences. However, none of these crossover pathways could be identified as the dominant contributor in either operating mode, primarily due to the low current density, where H_2_ crossover remains less pertinent. While it was impossible to mitigate the H_2_ loss/crossover for the liquid-fed anode bioelectrochemical experiments, we slowed down the H_2_ crossover during the vapor-fed anode experiment by pressurizing the anode compartment to 100 mBar. However, this did not improve electromethanogenesis.

A major contributor to performance loss is the poisoning of the catalyst layer itself. While the Htec-PFSA ion-exchange membrane generally underperformed, the Nafion 117 experiments showed stable H_2_ production in both liquid- and vapor-fed anode operating modes. The difference between the modes is the increased cell voltage, where the vapor-feeding shows its major benefit with its lower and more stable cell voltage during the first 42 days. Several studies have shown that catalyst poisoning is a major contributor to decreased current densities in which the catalyst layer is blocked by precipitate or the constant electrolyte flow causes abrasion.^8^ The catalyst itself can react with the electrolyte to deactivate its catalytic ability. Here, Pt could have been poisoned through sulfide that adsorbs on the active sites of Pt and inhibits the H_2_ evolution reaction.^27^ Other elements that might have been electroplated on the cathode layer showed no concentration reduction except for Ni, which is needed for the methanogen to grow. The other measured elements were protected by a chelating agent (NTA). de Smit et al. showed that metals electroplated less on the cathode when a chelating agent was used.^5^ We introduced the PTFE membrane to protect the catalyst, and only small improvements were observed in a lower and more stable cell voltage. It is, therefore, necessary to further protect the catalyst layer by separating it better from the electrolyte, such as different hydrophobic membranes, or using an improved catalyst inert to the electrolyte.

NiMo has been shown to be stable with methanogen medium while having the same catalytic prowess as Pt.^28^^,^^29^ A plate reactor design utilizing NiMo as cathode catalysts demonstrated current densities comparable to those observed in this study.^13^ Pentlandite-type catalysts (Fe_3_Co_3_Ni_3_S_8_) also show good catalytic activity and biocompatibility with elevated stability in the presence of H_2_S.^8^ Rad and colleagues (2023), who used pentlandite in their hybrid zero-gap bioelectrochemical cell, showcased high current densities. However, they protected their catalysts with a porous transport layer, which is composed of a PTFE-coated titanium mesh and is commonly used in abiotic electrolyzers to protect the catalysts layer.^8^ Similar to their study, we protected the Pt catalyst with a PTFE layer. The performance decreased once the cell voltage increased to higher cell voltages that might have corroded the anode catalyst. In our opinion, the idea of bioelectrochemistry is not to protect the catalyst with a hydrophobic layer to prevent contact between the catalyst layer and electrolyte and to have the miscible H_2_ directly uptaken by the microbes. Here and elsewehere,^8^ a PTFE layer protected the catalysts. In this case, is microbial electrosynthesis even an option? If high current densities can only be achieved by including a hydrophobic layer between the catalyst layer and electrolyte, an abiotic electrochemical cell can be used instead, and the evolved H_2_ gas can be transported to the bioreactor through a hydrophobic membrane to generate smaller H_2_ bubble size, avoiding all the constraints of bioelectrochemical processes. However, a yet less explored prospect in microbial electrochemistry is using a biofilm at which high current densities without protecting the electrode are theoretically possible.^30^ While creating a biofilm with a high surface area cathode, current densities more than 10 mA cm^−2^ were possible, however, with cell voltages of more than 3 V.^7^

In theory, the benefit of using vapor-fed anode bioelectrochemical cells is the circumvention of a pH gradient that alters the thermodynamic cell potential of water splitting. Ideally, the water vapor at the anode is split into O_2_ and protons. The protons are then transported to the cathode to form H_2_, leaving the anode compartment at a neutral pH.^31^ Our study showed no improvement in the cell voltage during the vapor-fed anode experiment compared to the liquid-fed anode experiment. Both operating modes had similar starting cell voltages, which diverged to similar final higher cell voltages. The supposedly faster degradation of the electrocatalysts for the liquid-fed anode experiment compared to the vapor-fed anode experiment makes vapor-feeding a better option to protect the catalysts from, for example, abrasion. The abiotic experiment indicated that water accumulation between the cathode and the PTFE membrane likely led to the formation of a pH gradient, resulting in an increase in cell voltage. However, a proof of circumventing a pH gradient by using a vapor-fed anode could not be observed.

### Limitations of the study

This study presents a potential approach for the industrial application of microbial electrosynthesis, with a techno-economic analysis needed to assess its feasibility. However, a considerable challenge remains in the form of low current density and the consequent low EE. Jourdin et al. (2020) postulated that microbial electrosynthesis becomes economically feasible only when the EE exceeds 50%.^32^ Here, the peak EEs recorded were 44.4% for liquid-fed anode and 39.7% for vapor-fed anode experiments (Figures 1B and 2B; Table S2), falling short of the desired threshold. An EE of 50.2% was reached when the cathode was protected with a PTFE layer. A contributing factor to this inefficiency is the excess H_2_ in the headspace. Recirculating the headspace gas could increase the amount of soluble H_2_ and increase the EE to more than 50%, making microbial electrosynthesis an option for industrial applications.^10^ Moreover, vapor-fed anode electrolyzers encounter a specific limitation concerning the water content within the membrane.^31^ Low water content in the membrane can lead to increased ohmic resistance. In our experiments, we consistently supplied water vapor to the anodic compartment to maintain high humidity levels inside the cell. However, the humidity levels were not measured, which should be the aim of future studies. Lastly, our study did not perform replicates due to the engineering development aspect of this work, including trial and error. However, we acknowledge this limitation, and future studies should incorporate replicates to strengthen the reliability and reproducibility of the findings.

## Resource availability

### Lead contact

Further information and request for resources should be directed to the lead contact, Largus T. Angenent (l.angenent@uni-tuebingen.de).

### Materials availability

This study did not generate new unique materials.

### Data and code availability

All datasets are available in the manuscript or supplemental information. The paper does not report original code. Any additional information reported in this paper is available from the lead contact upon request.

## Acknowledgments

This work was supported by the 10.13039/501100001659Deutsche Forschungsgemeinschaft (DFG, German Research Foundation, EBiotech, SPP2240; L.T.A.)—project number 445506379, the 10.13039/100005156Alexander von Humboldt Foundation in the framework of the Alexander von Humboldt Professorship (L.T.A.), and The 10.13039/501100009708Novo Nordisk Foundation CO_2_ Research Center with grant number NNF21SA0072700 (L.T.A.). We acknowledge support from the Open Access Publication Fund of the University of Tbingen. We want to thank the anonymous reviewers for improving the manuscript considerably.

## Author contributions

N.R., methodology, formal analysis, investigation, data curation, writing—original draft and review and editing, visualization, and funding acquisition; L.T.A., conceptualization, resources, writing—review and editing, supervision, project administration, and funding acquisition.

## Declaration of interests

The authors declare no competing interests.

## STAR★Methods

### Key resources table

**Table:** 

REAGENT or RESOURCE	SOURCE	IDENTIFIER
**Bacterial and virus strains**		

*Methanothermobacter thermautotrophicus* ΔH	DSMZ	DSM 1053

**Software and algorithms**		

OriginPro 2024	OriginLab Corporation, Northampton, MA, USA	https://www.originlab.com/
EC-Lab	Bio-logic	N/A
Peaksimple version 4.88	SRI Instruments, Earl St. Torrance, CA, US	N/A

**Other**		

Electrochemical cell	H-tec Education	E208
Coated Nafion 117 membrane	Quintech	CCM-E25-N117
Ti-felt	Quintech	11675
Coated carbon paper	Quintech	GDL240
PTFE membrane	Bola	N1617-10
Potentiostat	Bio-logic	VMP3
MilliGas counter	Dr.-Ing. RITTER Apparatebau GmbH & Co. KG	MGC-1 V3.4 PMMA
Bronkhorst EL-Flow® Prestige mass flow controllers	Bronkhorst Deutschland Nord GmbH	https://www.bronkhorst.com/int/products/gas-flow/el-flow-prestige/

### Experimental model and study participant details

#### Strain and medium

*M. thermautotrophicus* ΔH (DSM 1053) was purchased from DSMZ (Braunschweig, Germany). The medium was adjusted from Martin et al.^21^ and contained (*per* liter): KH_2_PO_4_, 1.36 g; sodium sulfate, 0.71 g; trisodium nitrilotriacetate, 0.21 g; nitrilotriacetic acid (NTA), 0.076 g; ammonium nickel(II) sulfate, 1.97 mg; cobalt(II) sulfate heptahydrate, 0.71 mg; sodium molybdate dihydrate, 0.61 mg; magnesium sulfate heptahydrate, 0.25 mg; iron(II) sulfate, 0.05 g; ammonium sulfate, 0.06 g; sodium selenate, 0.26 mg; and sodium tungstate dihydrate, 0.32 mg. *M. thermautotrophicus* ΔH was cultivated for the inoculum in serum bottles containing 6 g/L sodium bicarbonate as a pH buffer and 0.5 g/L L-cysteine hydrochloride as a reducing agent and sulfur source. The serum bottles were sparged with N_2_/CO_2_ (80/20%, v/v) to create an anoxic environment. Subsequently, the headspace was replaced with H_2_/CO_2_ (80/20%, v/v) at 1 bar overpressure and autoclaved. Incubation of *M. thermautotrophicus* ΔH occurred at 60°C and 150 rpm. The bioelectrochemical system underwent a similar anoxic treatment by sparging with pure CO_2_ before supplementation and inoculation. Following the establishment of anoxic conditions, the sulfur source (0.3 g/L sodium sulfide) was added, and the pH was adjusted to 7.2 using 1M NaOH, which was diluted in the medium instead of water.

### Method details

#### Bioelectrochemical setup

The electrochemical cell was a commercially available zero-gap electrolysis cell (Figure S6B and S6C, E208, H-tec Systems GmbH, Augsburg, Germany) composed of a membrane electrode assembly. The perfluorosulfonic acid (PFSA) ion-exchange membrane was coated with 0.5 mg cm^−2^ Pt on the cathode side and 2 mg cm^−2^ IrRu mix on the anode side. A carbon paper with a mesoporous carbon layer and Ti-felt was placed on top of the catalyst and contacted by the current collector. In subsequent experiments, we only kept the frame of the electrolysis cell. However, the catalysts-coated ion-exchange membrane and the gas diffusion layer were exchanged with a Nafion 117 ion-exchange membrane coated with 1 mg cm^−2^ Pt/C on the cathode and 2 mg cm^−2^ Ir on the anode (CCM-E25-N117, Quintech, Göppingen, Germany). The gas diffusion electrodes were carbon paper with a mesoporous layer and Ti-felt for the cathode and anode side (Quintech, Göppingen, Germany), respectively. A PTFE membrane (N1617-10, Bola, Bohlender GmbH, Grünsfeld, Germany) was used to protect the cathode electrocatalyst. The total geometric surface area of the zero-gap bioelectrochemical cell was 16 cm^2^, and a constant current of 120 mA (7.5 mA cm^−2^) or 400 mA (25 mA cm^−2^) was applied *via* a potentiostat (VMP3, Bio-logic, Claix, France) and controlled through EC-lab software (Bio-logic, Claix, France). The anode compartment and cathode compartment (with a volume of 4 mL) were connected to glass recirculation vessels with a similar volume of 300 mL (Figure S6A), and the recirculation rate for both the anolyte and catholyte was 25 mL min^−1^. The catholyte glass recirculation vessel contained multiple ports for pH measurement, gas feed, outgas, in/out ports for the glass recirculation, gas sampling, base feed for pH control, medium feed, and medium out. The anolyte recirculation vessel only contained ports to monitor the pH and recirculate the water to the electrochemical cell.

The anolyte and catholyte were maintained at 65°C in the vessels using a water thermostat (KISS 104A, Huber, Raleigh, USA). During vapor-fed anode operation, the anolyte was replaced by humid air. Humid air was generated by heating water to 65°C and applying a N_2_ gas flow rate of 3 mL min^−1^ (6 mL min^−1^ when the PTFE membrane was installed). A multi-channel pump (Masterflex L/S pump equipped with a multi-channel pump head model 7519-20, Cole Parmer, Germany) was used as medium feed in and out during continuous operation (Figure S6A). CO_2_ was used as a carbon source, and the flow rate was controlled using the Bronkhorst EL-Flow® Prestige mass flow controller (Bronkhorst Deutschland NORD GmbH, Kamen, Germany). The CO_2_ flow rate was adjusted from 0.6 mL min^−1^ to 0.3 mL min^−1^ to ensure that carbon was not limited. The outgas volume was measured offline using a MilliGascounter MGC-1 V3.4 PMMA (Dr.-Ing. RITTER Apparatebau GmbH& Co. KG, Bochum, Germany).

Before inoculation, the redox potential of the catholyte medium was reduced with 0.3 g L^−1^ sodium sulfide, and the pH was set to 7.2. For the first experiment, *M. thermautotrophicus* ΔH pre-cultures grown in serum bottles were used to inoculate the bioelectrochemical cell to an initial OD_600_ of approximately 0.04 (1% v/v). All subsequent experiments were inoculated using biomass from this initial culture harvested at the end of the liquid-fed anode with the Htec-PFSA membrane experiment. Three experiments were conducted with different operating conditions. The first experiment was the commercial electrochemical cell with MilliQ water as the anolyte. The second experiment replaced the ion-exchange membrane with the Pt/Ir-coated Nafion 117 and used MilliQ water as the anolyte. The third experiment was similar to the second experiment, except with vapor feeding the anode side. The cathodic vessel was first sparged with pure CO_2_ for each experiment to make the catholyte/medium anoxic. The fourth experiment was a replication of the third experiment with including a PTFE membrane.

The bioelectrochemical cell was operated in fed-batch mode for the entire operating period of each experiment except during continuous mode during days 52–80 (third experiment). At the start, 300 mL of 1x medium was used for the catholyte. After two weeks, 3 mL of a 100x medium was added. 0.5 mL of 40 g L^−1^ Na_2_S was added every week. The continuous mode operation was exclusively applied to the third experiment. It was initiated only after completing the fed-batch phase to compare it with the liquid-fed anode bioelectrochemical cell experiments. The hydraulic retention time (HRT) was 7 days, corresponding to a constant feed rate of 0.03 mL min^−1^. The pH of the anolyte and catholyte was monitored (Bluelab, Gisborne, New Zealand) with a pH electrode (662–1767, VWR, Darmstadt, Germany). A sample day consisted of measuring OD_600_, pH, and out gas composition. The liquid sample was stored at −20°C until further analysis. The ohmic resistance of the zero-gap bioelectrochemical cell was measured using the current interrupt method using the potentiostat (Bio-logic, Claix, France). A current of 120 mA or 400 mA, which was the applied current during the zero-gap bioelectrochemical experiments, was used during the current interrupt with 1-s intervals repeated 10 times at abiotic conditions.

#### Analytical analysis

A gas chromatograph (SRI GC, Torrance, USA) was used to analyze the headspace gas composition. The gas chromatograph was equipped with a Haysep D column (length 3m, outer diameter 1/8″, SRI GC). A thermal coupled detector and a flame ionization detector were used to measure H_2_, CH_4_, and CO_2_ with N_2_ as the carrier gas. The column temperatures and pressures were set at 70°C and 20 psi, respectively. 500 μL was taken from the gas headspace with a gastight syringe (Hamilton, Reno, USA) for each sample point. The OD_600_ was measured with a UV/VIS-Spectrophotometer at 600 nm absorbance (BioMate™ 160, ThermoFischer Scientific, Waltham, USA). All liquid samples were analyzed on an Agilent 7900 ion-coupled plasma mass spectrometer instrument (Agilent, Santa Clara, USA) to quantify ^23^Na, ^24^Mg, ^39^K, ^44^Ca, ^55^Mn, ^56^Fe, ^59^Co, ^60^Ni, ^66^Zn, ^78^Se, and ^95^Mo. The samples were diluted 1:100 in 1% HNO_3,_ which was also the matrix.

### Quantification and statistcal analysis

#### Plotting data

All graphs were created in OriginPro 2024.

#### Calculations

Coulombic efficiency was calculated by dividing the produced CH_4_ or H_2_ in number of electrons by the number of electrons supplied to the electrochemical cell. The equation is as follows:

(Equation 1) $$\text{CE}=\frac{n\cdot F\cdot z}{\int \text{Idt}}$$

Where n is the molar quantity of produced CH_4_ or H_2_, F the Faraday constant, z number of electrons to form CH_4_ or H_2_, and I the current of the bioelectrochemical cell. The energy efficiency was calculated as follows:^33^

(Equation 2) $$\text{EE}=\frac{\Delta G_{\text{CH}4}\cdot n}{E_{\text{cell}}\cdot \int \text{Idt}}$$

With ΔG_CH4_ the Gibbs free energy of CH_4_ (890.4 kJ/mol), and E_cell_ the measured cell voltage.

The conversion efficiency of H_2_ to CH_4_ was calculated as follows:

(Equation 3) $$\text{Conversion}\;\text{efficiency}\;H_{2}\;\text{to}\;{\text{CH}}_{4}=\frac{{4n}_{{\text{CH}}_{4}}}{{4n}_{{\text{CH}}_{4}}+n_{H_{2}}}$$

With n_CH4_ the quantity of mole of CH_4_ produced and n_H2_ the quantity of mole of H_2_ in the off-gas.
