# Supplementary material for: A development study for liquid- and vapor-fed anode zero-gap bioelectrolysis cells
Source: iScience. 2025 Jun 19;28(7):112959. doi: 10.1016/j.isci.2025.112959 (PMC12272894; doi:10.1016/j.isci.2025.112959)
Supplement: Document S1. Figures S1–S6 and Tables S1 and S2 [file mmc1.pdf]

iScience, Volume *28*

Supplemental information

A development study for liquid- and vapor-fed anode zero-gap bioelectrolysis cells

Nils Rohbohm and Largus T. Angenent

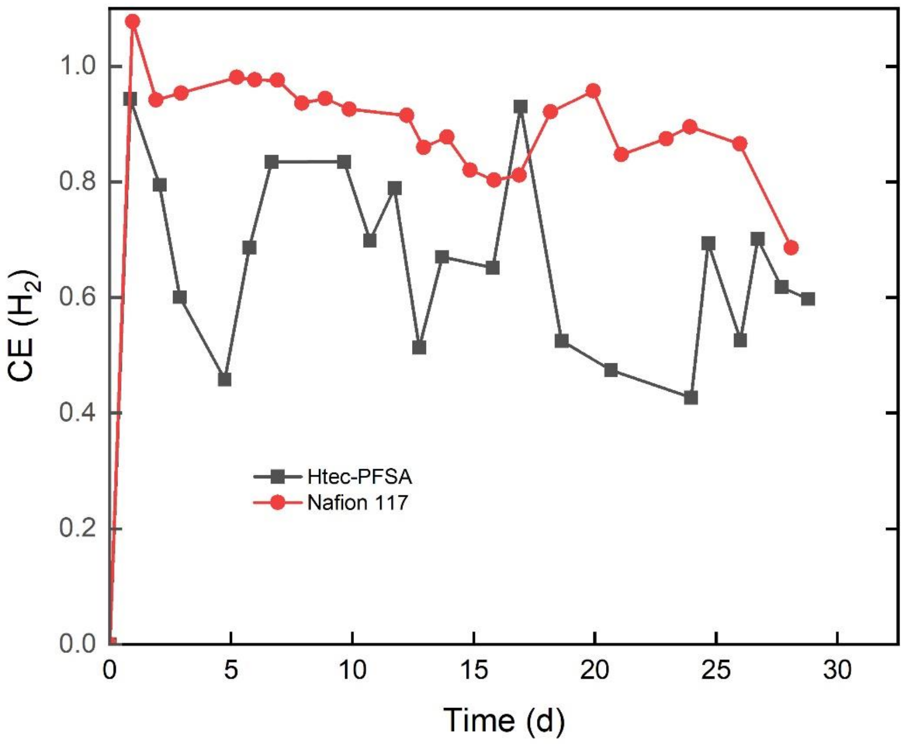

Figure S1: CE of H_2 during the liquid-fed anode zero-gap bioelectrochemical cell experiment using Htec-PFSA (dark grey) and Nafion 117 (red) as ion-exchange membrane.

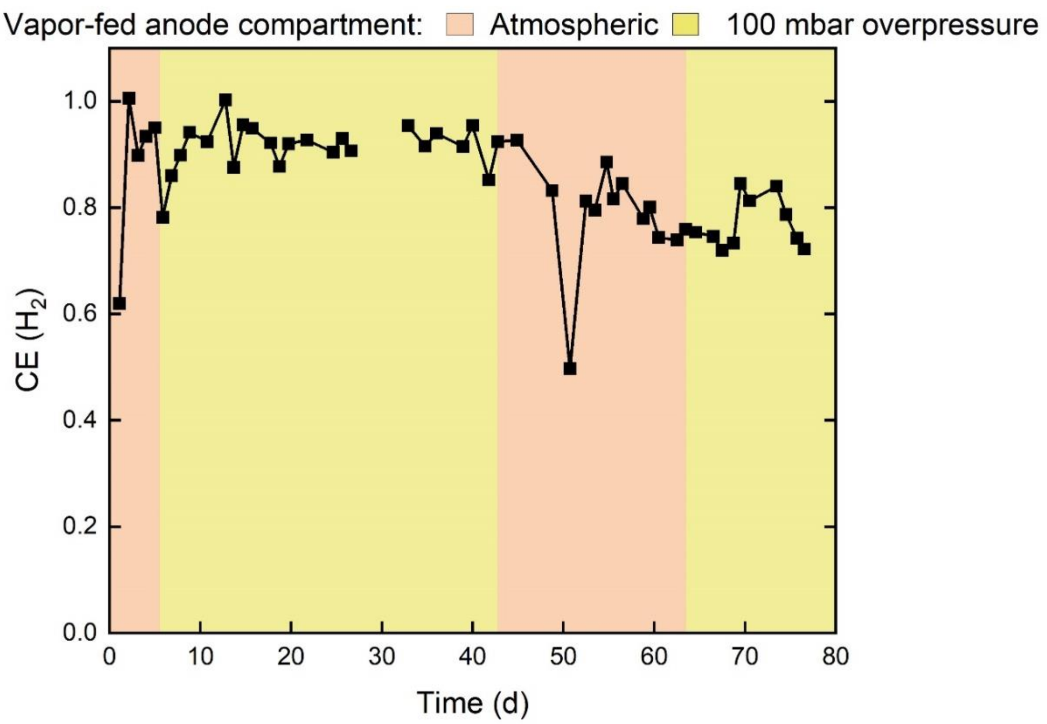

Figure S2: CE of H_2 during the vapor-fed anode zero-gap bioelectrochemical cell experiment using Nafion 117 as the ion-exchange membrane. The anode compartment of the bioelectrochemical cell was kept at atmospheric pressure (orange) or 100 mbar overpressure (yellow).

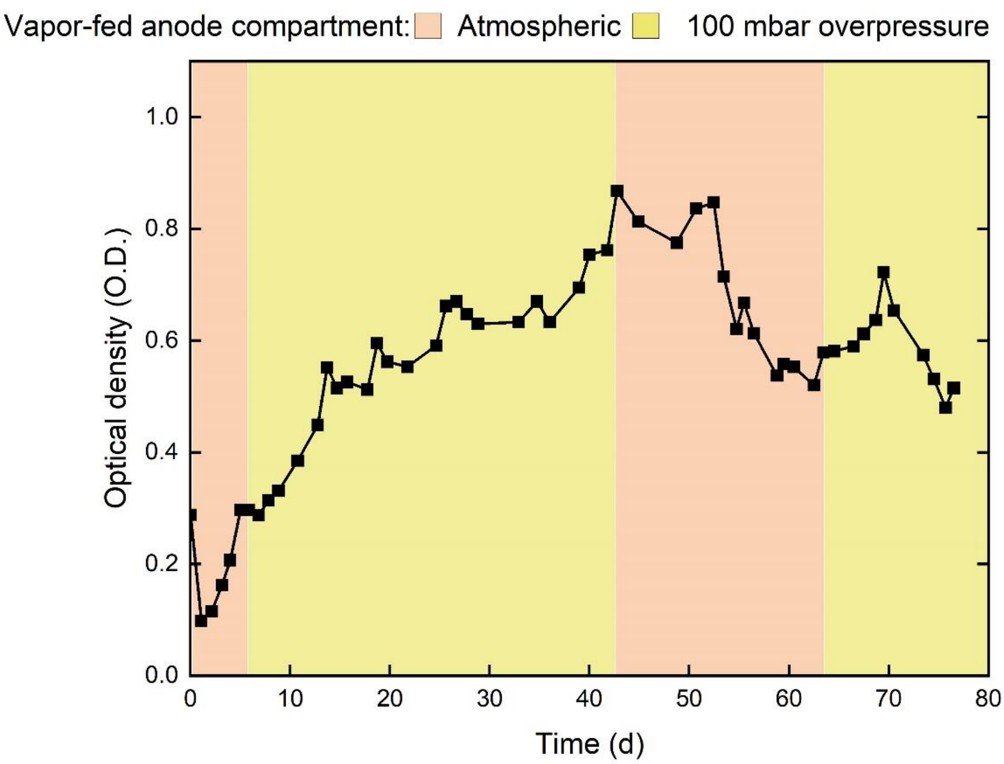

Figure S3: optical density (O.D.) during the vapor-fed anode zero-gap bioelectrochemical cell experiment using Nafion 117 as the ion-exchange membrane. The anode compartment of the bioelectrochemical cell was kept at atmospheric pressure (orange) or 100 mbar overpressure (yellow).

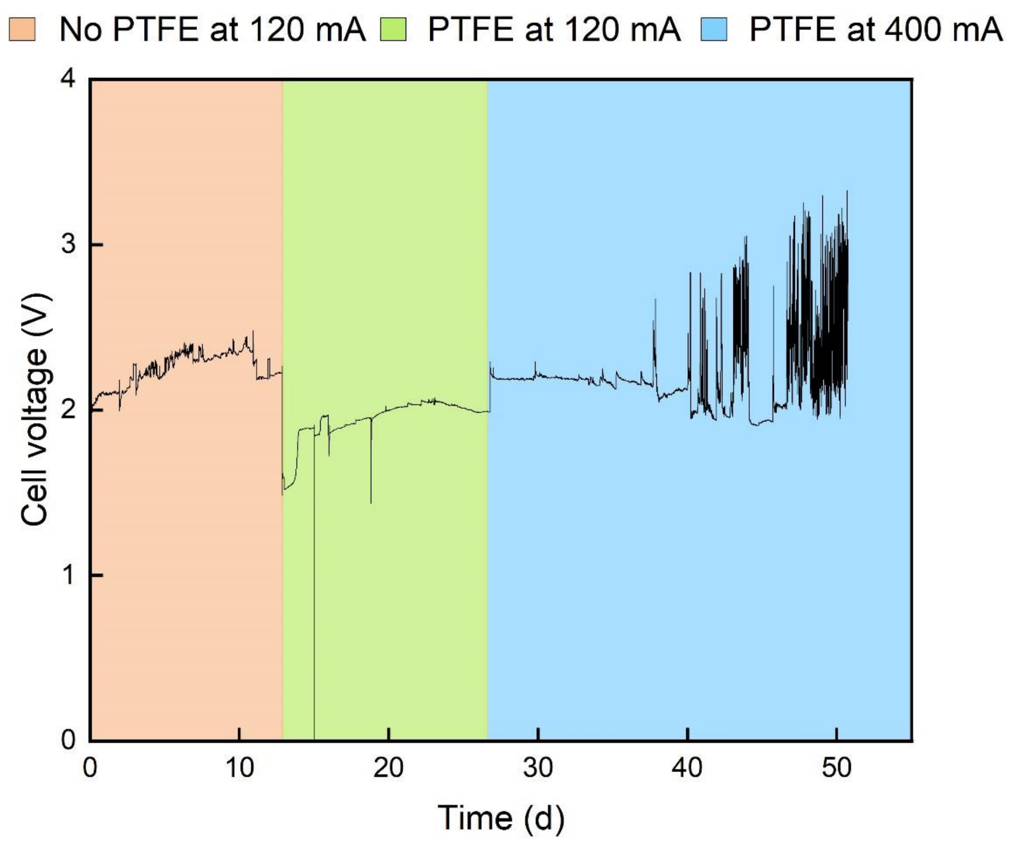

Figure S4: cell voltage of the vapor-fed anode bioelectrochemical cell which was run abiotically at 120 mA (green) and 400 mA (blue). The orange phase represents the initial operation of the bioelectrochemical cell without a PTFE membrane, while the green phase signifies the start of operation with the PTFE membrane added. In the blue phase, the current was increased from 120 mA to 400 mA with the PTFE membrane in place.

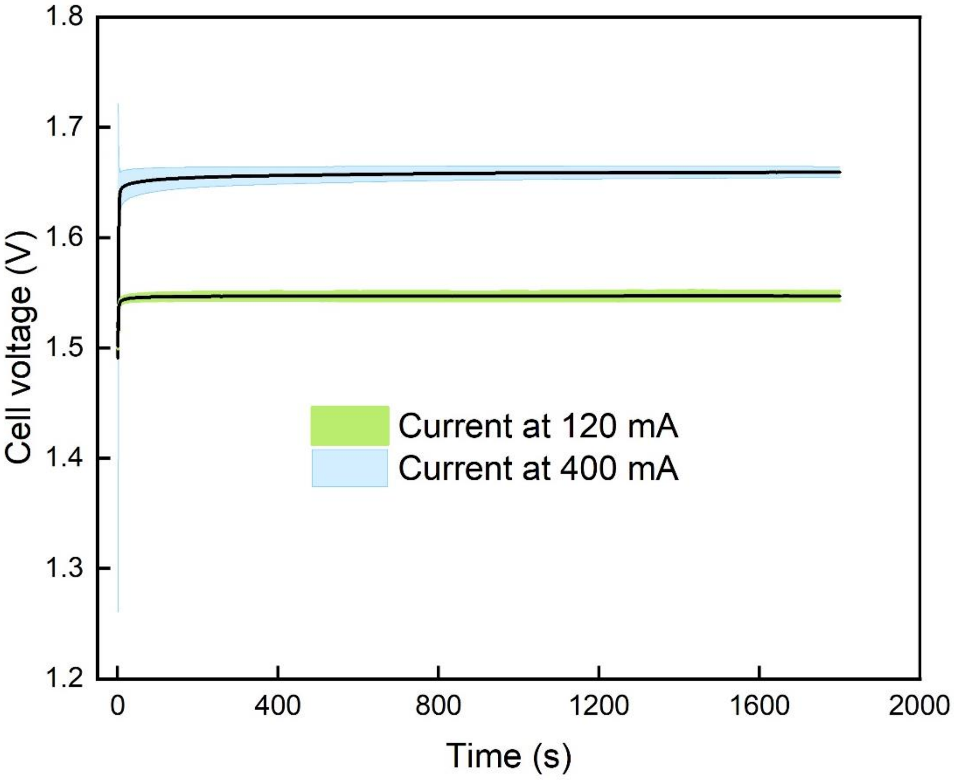

Figure S5: cell voltage of the vapor-fed anode bioelectrochemical cell which was run abiotically at 120 mA (green) and 400 mA (blue).

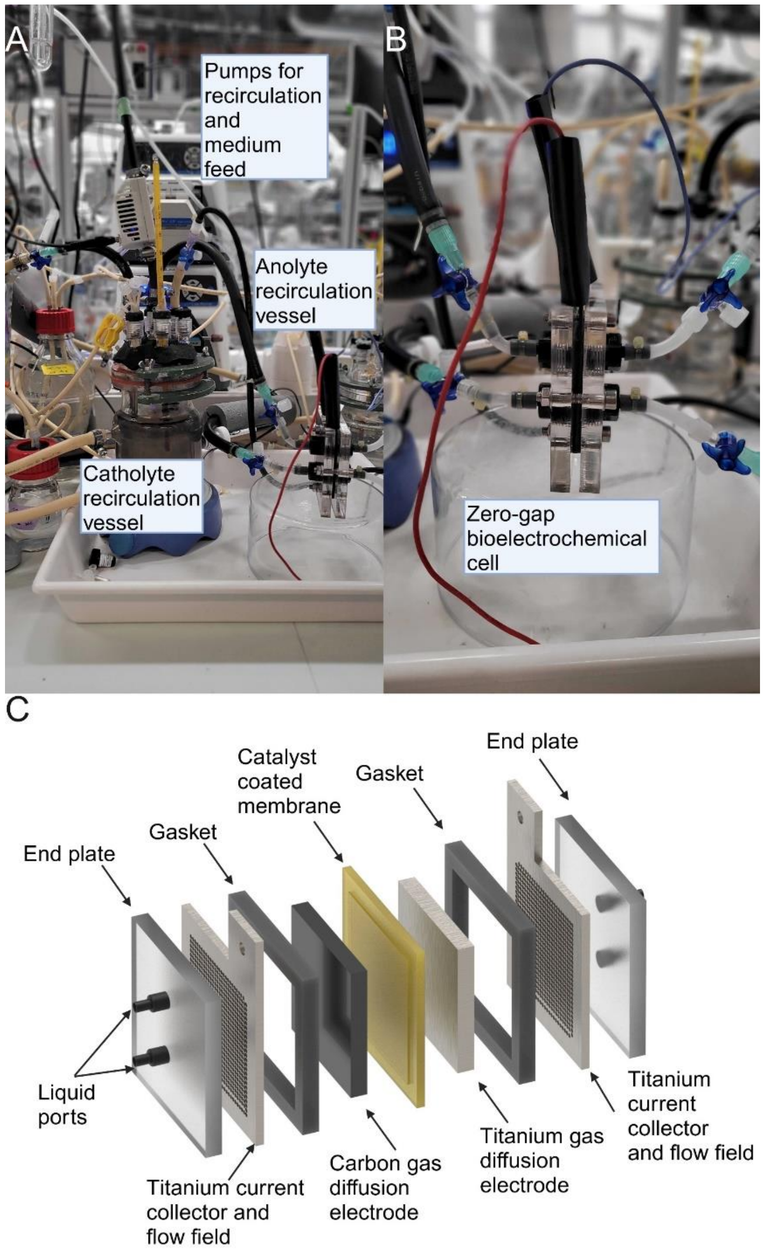

Figure S6: Lab view of the zero-gap bioelectrochemical cell. (A) Image of the zero-gap bioelectrochemical cell in operation; (B) Close-up view of the zero-gap bioelectrochemical cell; and (C) exploded view of the zero-gap bioelectrochemical cell. The catholyte glass recirculation vessel contained ports to monitor and control the pH and to sample gas. Two ports were used to recirculate the electrolyte/medium. Liquid samples were taken from a three-way valve at the recirculation tubing line. Two ports were used for the gas-in and the gas-out. The gas-out tubing line contained a condenser to avoid water loss. The ports for the anolyte glass recirculation vessel consisted of the recirculation tubing and a pH probe for pH monitoring. During the vapor-fed anode experiment, ports for N_2 gas-in and a tubing connected to the anodic compartment were used.

Table S1 | Performance data of published bioelectrochemical systems for microbial electromethanogenesis.

Reference	Reactor Type	Inoculum	Operating days	Cell voltage (V)	Current density ($mA\ cm^{-2}$)	Geometric CH_4 production rate ($L\ m^{-2}\ d^{-1}$)	Reactor volumetric CH_4 production rate ($L\ L^{-1}\ d^{-1}$)	Cathode compartment volumetric production rate ($L\ L^{-1}\ d^{-1}$)	Coulombic efficiency (%)	Energy efficiency (%)	CH_4 production efficiency ($L\ kWh^{-1}$)
Geppert et al. (2019)[1]	Redox-flow battery	Biogas plant	1	-	3.5	62.5	12.5	2.7	-	-	-
			5	-	2.5	47.5	9.5	2.1	-	30	
Cai et al. (2022)[2]	Moving bed biofilm	Sludge	7	5.5	11.1[c]	141.0[c]	1.4[c]		46	-	9.5[b]
Baek et al. (2022)[3]	Zero-gap	Sludge	6	3.1	1.7	16.7[b]	-	2.9	-	-	0.01[b]
Shang et al. (2023)[4]	CSTR with tubular electrolyzer	Sludge	21	6	11.9	317.8	0.5	-	98	20[c]	17.3[b]
Rad et al. (2023)[5]	Zero-gap	Sludge	11	2.1 to 2.3	30	622	2.4	-	83	43	39.2[b]
Rohbohm et al. (2024)[6]	Flow field	*Methanothermobacter thermautotrophicus*	125 (Nickel experiment)	3	3.8[c, d]	90.7[c, d]	2.6[c, d]	15.1[c, d]	94.7[c, d]	36.4[c, d]	32.9[d]
Lavender et al. (2025)[7]	Tubular with a bubble column	Anaerobic granular sludge	470	2.6	0.6	15.3[b]	0.28[b]	-	88	40	36.8[b]
Deutzmann et al. (2025)[8]	Flat plate	*Methanococcus maripaludis*	~32[a]	3.0[a]	6.3	89[c]	8.9[c]	14[c]	59[c]	24[c]	19.6[b]
			~88[a]	2.7[a]	4.6	108[c]	10.8[c]	17[c]	78[c]	30[c]	36.2[b]
Jayathilake et al. (2025)[9]	Bubble column	*Methanococcus maripaludis*	60	2.9	0.6	-	1.0	4.0	99	42	35.9[b]
			12	3.3[a]	1.3	-	3.4	12.8	96	35	42.9[b]

[a]taken from figure

[b]calculated based on the information of the publication

[c]maximum values

[d]taken from the authors

Table S2 | Performance data of the zero-gap bioelectrochemical systems from this study.

Zero-gap setup	Operating days	Cell voltage (V) range (cell voltage in V used for EE)	Absolute current in mA (current density in mA cm^{-2})	Maximum geometric CH_4 production rate in $L \cdot m^{-2} \cdot d^{-1}$	Maximum reactor volumetric CH_4 production rate in $L\ L^{-1}\ d^{-1}$	Maximum cathode compartment volumetric production rate in $L\ L^{-1}\ d^{-1}$	Maximum CH_4 Coulombic efficiency in %	Maximum energy efficiency in %	Maximum CH_4 production efficiency in $L\ kWh^{-1}$
Liquid-fed anode with Htec membrane	28	1.9 to 2.4 (2.3)	120 (7.5)	149.7	0.8	59.9	74.3	36.8	35.7
Liquid-fed anode with Nafion 117 membrane	28	1.8 to 2.3 (2.2)	120 (7.5)	170.0	0.8	68.0	84.3	44.4	43.1
Vapor-fed anode with Nafion 117 membrane	71	1.6 to 2.3 (2.2)	120 (7.5)	156.2	0.8	62.5	77.5	39.7	38.5
Vapor-fed anode with Nafion 117 membrane and PTFE membrane at 120 mA	13	1.6 to 2.0 (1.9)	120 (7.5)	152.9	0.8	61.1	75.8	46.6	45.4
Vapor-fed anode with Nafion 117 membrane and PTFE membrane at 400 mA	23	2.1 to 2.4 (2.0)	400 (25)	604.5	3.2	241.8	90.0	50.2	48.7

Supplementary reference

1. Geppert, F., Liu, D., Weidner, E., and ter Heijne, A. (2019). Redox-flow battery design for a methane-producing bioelectrochemical system. International Journal of Hydrogen Energy *44*, 21464 - 21469.
2. Cai, W., Cui, K., Liu, Z., Jin, X., Chen, Q., Guo, K., and Wang, Y. (2022). An electrolytic-hydrogen-fed moving bed biofilm reactor for efficient microbial electrosynthesis of methane from CO_2. Chemical Engineering Journal *428*, 132093. https://doi.org/10.1016/j.cej.2021.132093.
3. Baek, G., Rossi, R., Saikaly, P.E., and Logan, B.E. (2022). High-rate microbial electrosynthesis using a zero-gap flow cell and vapor-fed anode design. Water Research *219*, 118597. 10.1016/j.watres.2022.118597.
4. Shang, G., Cui, K., Cai, W., Hu, X., Jin, P., and Guo, K. (2023). A 20 L electrochemical continuous stirred-tank reactor for high rate microbial electrosynthesis of methane from CO2. Chemical Engineering Journal *451*, 138898. https://doi.org/10.1016/j.cej.2022.138898.
5. Rad, R., Gehring, T., Pellumbi, K., Siegmund, D., Nettmann, E., Wichern, M., and Apfel, U.-P. (2023). A hybrid bioelectrochemical system coupling a zero-gap cell and a methanogenic reactor for carbon dioxide reduction using a wastewater-derived catholyte. Cell Reports Physical Science *4*. 10.1016/j.xcrp.2023.101526.
6. Rohbohm, N., Lang, M., Erben, J., Gemeinhardt, K., Patel, N., Ilic, I.K., Hafenbradl, D., Rodrigo Quejigo, J., and Angenent, L.T. (2024). Performance Effects of Different Shutdown Methods on Three Electrode Materials for Electromethanogenesis. ChemElectroChem *11*, e202400372. https://doi.org/10.1002/celc.202400372.
7. Brandão Lavender, M., Steller, J., Liu, D., de Rink, R., Tofik, S., and ter Heijne, A. (2025). Designing, building and operating an up-scaled methane producing bioelectrochemical system for power-to-methane. Journal of Power Sources *629*, 236010. https://doi.org/10.1016/j.jpowsour.2024.236010.
8. Deutzmann, J.S., Callander, G., and Spormann, A.M. (2025). Improved reactor design enables productivity of microbial electrosynthesis on par with classical biotechnology. Bioresource Technology *416*, 131733. https://doi.org/10.1016/j.biortech.2024.131733.
9. Jayathilake, B.S., Chandrasekaran, S., Deutzmann, J.S., Kracke, F., Cornell, C., Worthington, M.A., Freyman, M.C., Jue, M.L., Spormann, A.M., Pang, S.H., and Baker, S.E. (2025). Additively Manufactured High Surface Area 3D Cathodes for Efficient and Productive Electro-Bio-Methanation. ACS Electrochemistry. 10.1021/acselectrochem.4c00109.
